# The Effect of Simulated Chewing on the Surface Roughness of Direct and Indirect Resin-Composites Opposed by Zirconia: An In Vitro Study

**DOI:** 10.1155/2022/8686540

**Published:** 2022-09-10

**Authors:** Ghadeer S. AlSuwaidi, Ruwaida Z. Alshali, Nesreen A. Salim, Julian D. Satterthwaite, Nick Silikas

**Affiliations:** ^1^Fixed and Removable Prosthodontics Department, Hamad Dental Services, Hamad Medical Corporation, Doha 00974, Qatar; ^2^Oral and Maxillofacial Prosthodontics Department, Faculty of Dentistry, King Abdulaziz University, P.O. Box 80200, Jeddah 21589, Saudi Arabia; ^3^Prosthodontic Department, School of Dentistry, The University of Jordan, Jordan University Hospital, Amman 11942, Jordan; ^4^Division of Dentistry, School of Medical Sciences, University of Manchester, Manchester M13 9PL, UK

## Abstract

**Purpose:**

To assess the surface roughness of two different light-cured resin-composites when opposed by monolithic zirconia after simulated mastication.

**Materials and Methods:**

Materials included a direct restorative nanohybrid (*n* = 10) and an indirect microhybrid (*n* = 10) resin-composite (Tetric EvoCeram and Sinfony, respectively). The antagonist material was 3 mol% yttria-stabilized tetragonal zirconia polycrystalline ceramic. Each material was subjected to *in vitro* chewing against zirconia using a chewing simulator for 250,000 cycles. A 3D profilometer was used to assess the surface roughness parameters of each resin-composite before and after the simulated chewing. Independent *t*-test and paired sample *t*-test were performed to compare roughness values for both materials and to compare baseline and after chewing simulation values (*p* = 0.05).

**Results:**

Sinfony showed significantly greater roughness values compared to Tetric EvoCeram (*p* ≤ 0.025) before and after simulated chewing, except for Sa and Sv parameters after simulated chewing where the difference between the two materials was insignificant (*p* = 0.06 and 0.89, respectively). Surface roughness increased for both materials after simulated chewing compared to baseline values, but the difference was insignificant (*p* ≥ 0.065). However, *Sa* (*p* = 0.04) and *Sv* (*p* = 0.012) for Tetric EvoCeram were significantly higher after compared to before chewing simulation.

**Conclusion:**

Tetric EvoCeram had a smoother surface than Sinfony before and after simulated chewing. Surface roughness for both materials was higher after simulated chewing compared to baseline values which represent surface damage that was significant for Tetric EvoCeram while Sinfony showed better resistance.

## 1. Introduction

An ideal dental restorative material should simulate natural teeth in terms of strength, esthetics, biocompatibility, and wear resistance [1]. Resin-composites and all-ceramic restorations are very popular due to their excellent aesthetic appearance and good mechanical properties [2]. The increased popularity of zirconium dioxide ceramics (zirconia) as a restorative material has resulted from their excellent mechanical properties, associated with advances in CAD/CAM technology and the development of milling techniques [3]. Zirconia exists in three different crystalline configurations according to the temperature range: monoclinic, from room temperature to 1170°C; tetragonal, from 1170°C to 2370°C; and cubic, at temperatures above 2370°C. The use of zirconia has expanded to include the fabrication of fully or partially sintered frameworks for porcelain veneered fixed restorations in addition to full coverage monolithic zirconia crowns without veneering porcelain [4, 5].

With advanced bonding systems, a high clinical success rate for resin-composites has been demonstrated with survival rate of 12 years [6]. Resin-composites are conservative, especially in tooth surface loss cases where providing full coverage restorations will further harm tooth structure and pulpal vitality [7], and are also cost-effective. Ideally, the restoration should remain smooth under mastication for a long period of time [8]; however, resin-composites are prone to surface roughness. Studies have shown that resin-composite can suffer a significant amount of wear if used to restore large cavities in molar areas especially for patients with parafunctional habits or if opposed by a rough surface antagonist [9, 10]. The smoothness of resin-composite is a critical factor for a successful restoration [11].

Rough surfaces can increase plaque accumulation resulting in periodontal disease, reduce restoration brightness, and increase the possibility of discoloration [12, 13]. Besides the effect on the optical properties and the periodontal health, surface roughness can influence mechanical properties by accelerating abrasion, decreasing resistance to wear, and reducing longevity of the restoration leading to mechanical failure [14]. Also, material characteristics such as the type of organic matrix, composition, and the size of filler particles among other factors will affect surface properties of resin-composites [15].

Resin-composites are usually used and light-cured intraorally to restore teeth. However, the direct application of these materials in deep cavities with insufficient enamel may lead to increased effect of polymerization shrinkage and marginal microleakage [16]. Therefore, laboratory fabricated indirect resin-composite materials are available. Theoretically, these should have superior clinical performance (due to superior mechanical properties); however, a systematic review comparing the longevity of direct and indirect materials in posterior teeth has shown contradictory evidence [17].

The wear of natural teeth and restorative materials is a complex and multifactorial phenomenon. Rough restorations may lead to accelerated wear due to an increase in the coefficient of friction [18]. Recently, studies revealed that polished zirconia exhibited a lower wear rate on enamel than other dental materials, such as metal alloy, veneering porcelain, and lithium disilicate [19, 20]. Also, it has been demonstrated that wear of opposing enamel by monolithic zirconia crowns after 6 months of clinical use is low and acceptable [21]. Moreover, recent studies have shown that zirconia and lithium disilicate result in less wear on opposing enamel compared to conventional feldspathic restorations [22, 23].

Modern technology has enabled simulation of human chewing cycle using chewing simulators that apply certain loads and frictional forces [24], with various methods available to assess surface roughness: qualitative and quantitative or contact and noncontact [25]. The most commonly used devices for surface profile measurements are laser reflectivity measuring systems, contact diamond, and noncontact laser modes [26]. As a less invasive technique, atomic force microscopy can be employed, which provides three-dimensional images at a nanometer resolution. In addition, it can be used for the measurement of stiffness, hardness, and modulus of elasticity of dental materials [27].

In the literature, many studies have reported on the wear effect of different restorative materials antagonizing enamel [5, 22, 28]. However, there is limited data describing the wear characteristics and interaction of different types of restorative materials opposing each other, representing a situation that is encountered in daily clinical practice when restoring dentitions. Since resin-composite materials have nowadays nearly replaced amalgam for restoring posterior teeth and are widely used for restoring anterior teeth, it would be of great clinical relevance to assess the surface changes and stability of this type of restorative material when opposed during function by the recently introduced considerably harder materials such as unveneered zirconia and other polycrystalline ceramics and to assess the suitability of this opposition. Thus, the aim of the current study was to investigate changes in surface roughness using a three-dimensional (3D) profilometer of a direct nanohybrid and indirect microhybrid commercially available resin-composites after antagonist wear against unveneered full contoured zirconia through a simulated chewing test. The null hypotheses were as follows: (i) there is no difference in surface roughness among the two tested resin-composite materials; (ii) there is no difference in surface roughness for each tested composite material after chewing simulation as compared with the baseline roughness values.

## 2. Materials and Method

### 2.1. Test Materials

The two resin-composite materials tested in this study were Tetric EvoCeram (Ivoclar Vivadent, Schaan, Liechtenstein), a direct nanohybrid material, and Sinfony (3M ESPE AG, Seefeld, Germany), a microhybrid indirect laboratory veneering composite. The zirconia ceramic was Lava Plus high translucency all-zirconia ceramic (3M ESPE, St Paul, MN, USA). The materials' type, composition, and manufacturer details are listed in Table 1. Sample size was calculated based on a standard deviation value of 0.04 *μ*m (as was shown in the data of a previous similar study) [9] with 80% power at a 5% significance level. Ten resin-composite specimens in each group were deemed appropriate.

### 2.2. Preparation of Specimens

#### 2.2.1. Zirconia Antagonist Specimens

The antagonist zirconia specimens (*n* = 20) were prepared by the manufacturer. These were rectangular blocks measuring 17 mm (length) × 7 mm (width) × 5 mm (height). The specimens were prepared by hand sawing, finished using a 1200-grit abrasive paper followed by coarse, medium, and fine diamond-impregnated silicone polishers (ZiLMaster Adjustment kits, Shofu Dental Gmbh) to get high gloss on both upper and lower sides.

The samples were polished with an electric contra-angle handpiece with water using the same intraoral polishing system (Dialite LD Extra-Oral Polisher System) designed for zirconia following the manufacturer's directions. The same operator performed all the polishing. The speed was set to manufacturer's recommendations of 8000 RPM. The same allotted time of 60 s per instrument was used. Medium and fine diamond polishing paste was used with a small round brush for 60 s with each grit.

#### 2.2.2. Resin-Composite Specimens

A custom mould was constructed of polytetrafluoroethylene (PTFE) material to produce rectangular resin-composite samples measuring 8 mm (width) × 7 mm (length) × 5 mm (height). The mould was open from the top and bottom.

Tetric EvoCeram (*n* = 10) was packed in the mould in increments of 1.5–2 mm thickness against an acetate matrix and a glass slab on the bottom to establish a flat surface, filled to slight excess, and covered with another acetate matrix strip and glass slab from the top. Each increment was cured using an Elipar S10 LED light (3 M ESPE, St Paul, MN) (wavelength 430–480 nm, intensity 1200 mW/cm^2^) for 20 s at a distance of 1.0 cm from the light curing tip.

Sinfony samples (*n* = 10) were prepared by packing the material into the mould in increments of 1.5-2 mm thickness that were initially polymerized using a Visio Alfa halogen light curing device (3M ESPE, St Paul, MN). Each increment was polymerized for 5 s at wavelength 400–500 nm and intensity of 400 mW/cm^2^ at a distance of 1.0 cm from the light curing source. The final polymerization was carried out in a Visio Beta light curing device (3M ESPE, St Paul, MN) using a program of light curing for 1 min followed by light curing for 14 min under vacuum at wavelength of 400–500 nm and intensity of 400 mW/cm^2^.

The top surface of each sample of both resin-composites was polished manually for 3 min using a Sof-Lex finishing and polishing system (3M ESPE, St Paul, MN). The system involves aluminium oxide discs (13 mm diameter) with four grits: course (100 *μ*m), medium (40 *μ*m), fine (24 *μ*m), and superfine (8 *μ*m). The discs were attached to a low-speed handpiece and were used sequentially to polish the samples starting with course and ending with superfine grit discs without water until the samples were visibly glossy. Polishing of all samples was performed with a single operator.

### 2.3. Mounting of the Specimens and Chewing Simulation

Each Lava Plus zirconia specimen was mounted in a metal antagonist holder using cold-cure acrylic resin. The samples were levelled parallel to the holder and mounted about 3 mm higher to allow uniform contact of the lower samples. A similar arrangement was used for the resin-composite, which was mounted in the lower sample holder using dental gypsum plaster type II, and about 3 mm high, and levelled using a putty index.

**Figure 1:**
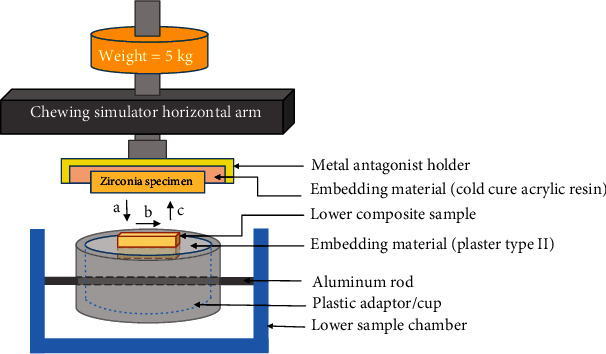
The specimen chamber and upper antagonist holder assembly of the chewing simulator. The loading cycle consists of (a) 2.5 mm vertical movement followed by (b) 0.7 mm horizontal movement then(c) an upward vertical movement.

The chewing simulation test was performed using the chewing simulator CS–4.2 (SD Mechatronik GmbH, Feldkirchen–Westerham, Germany). It is a dual axis chewing simulator that has been used to simulate two-body wear of dental materials [26]. Each chamber consisted of a plastic adapter or cup, a transparent cylinder and an o-ring. The samples were embedded in the plastic adapter fixed to the sample chamber by an aluminium rod. The whole assembly could be moved to the transverse direction by a butterfly screw engaged to the aluminium rod. The antagonist holder on the upper element of the chewing simulator allowed the specimen to be fastened using a screw. The upper element carrying the antagonist was able to travel in a downward, upward, or lateral direction, simulating chewing motion. The mounting of the specimens in the chewing simulator is illustrated in Figure 1.

A total number of 250,000 cycles were applied, allowing a combination of horizontal and vertical movement of the antagonist specimens. A load weight of 5 kg was exerted on each sample, applied vertically with a frequency of 1.6 Hz. The simulated chewing was carried out in a dry environment at room temperature. Chewing simulator parameters are summarised in Table 2.

### 2.4. Surface Roughness Measurements

A three-dimensional noncontact single-point sensor profilometer (Talysurf CLI 1000, Taylor-Hobson, Leicester, UK) with chromatic length aberration (CLA) gauge at 300 *μ*m was used to determine the degree of surface roughness of the resin-composites before and after chewing simulation. Before the chewing simulation, the resin-composite specimens were placed on the scanning table and scanned with a spacing of 10 *μ*m on the *x*-axis and 15 *μ*m on the *y*-axis. The scanning area was set at 5 mm on both *x*- and *y*-axes with a resolution of 501 and 334 points/traces on the *x*- and *y*-axes, respectively. The scanning distance on the *z*-axis was variable depending on the surface topography of each sample. The scanning speed was 500 *μ*m/s, and the average time for scanning was 61 min per specimen.

### 2.5. Surface Roughness Analysis

To analyse the scanned images of resin-composite specimens, the Talysurf CLI 1000 was connected to a control unit and a computer equipped with the TalyMap surface analysis software (Version 5.1, Taylor-Hobson, Leicester, UK) providing several parameters to assess surface topography. Four surface roughness amplitude parameters by ISO 25178 were selected in this study (*Sa*, *Sq*, *Sp*, and *Sv*), and the smaller the values, the smoother the surface. Surface texture parameters are described in Table 3.

### 2.6. Statistical Analysis

Statistical analysis was performed using SPSS for Windows release 22 (SPSS Inc., Chicago, IL, USA). The data were assessed for normality using the Shapiro-Wilk test, and all parameters were shown to be normally distributed (*p* ≥ 0.094). The mean and standard deviation for each parameter were calculated. Paired sample *t*-test was performed to compare the mean value for each parameter before and after chewing simulation. Independent *t*-test was performed to assess differences in roughness parameters between the two materials. The significance level was set at *p* < 0.05.

## 3. Results

**Figure 2:**
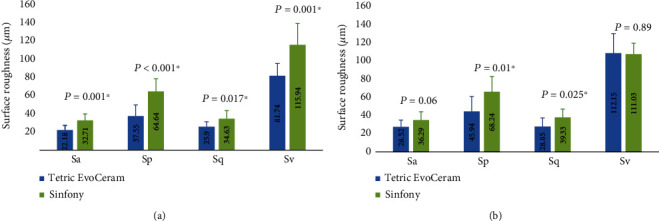
Bar charts representing the mean values of different surface roughness parameters before and after chewing simulation for the two resin-composite materials: (a) before chewing simulation and (b) after chewing simulation (*α* = 0.05). Error bars represent the standard deviation.

**Figure 3:**
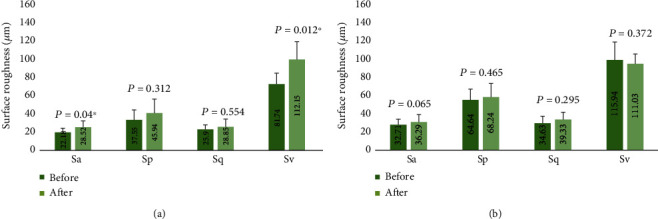
Bar charts representing the mean values of different surface roughness parameters of each resin-composite material before and after chewing simulation: (a) Tetric EvoCeram and (b) Sinfony (*α* = 0.05). Error bars represent the standard deviation.

Sinfony showed higher mean values for all surface roughness parameters when compared to Tetric EvoCeram, both before and after chewing simulation, except the *Sv* parameter after chewing simulation (Figure 2). Surface roughness values were also higher after chewing simulation when compared to before chewing simulation for both materials except *Sv* for Sinfony (Figure 3).

The independent sample *t*-test comparing the two materials against each other before and after chewing simulation showed that Sinfony had significantly greater roughness values compared to Tetric EvoCeram (*p* ≤ 0.025) except for *Sa* and *Sv* values after chewing simulation where the difference was insignificant (*p* = 0.06 and 0.89, respectively).

The paired sample *t*-test showed no significant difference in surface roughness parameters for each resin-composite when baseline values were compared to after chewing simulation values (*p* ≥ 0.065) except *Sa* (*p* = 0.04) and *Sv* (*p* = 0.012) for Tetric EvoCeram which were significantly higher after chewing simulation.

The percentage increase in surface roughness was generally higher for Tetric EvoCeram compared to Sinfony for all parameters except *Sq* where Sinfony showed a higher value (Table 4). The percentage increase of roughness for all parameters between the two resin-composite materials was higher for Tetric EvoCeram although not statistically significant (*p* ≥ 0.14) except for *Sv* where Tetric EvoCeram showed significantly higher value compared to Sinfony (*p* = 0.006).

**Figure 4:**
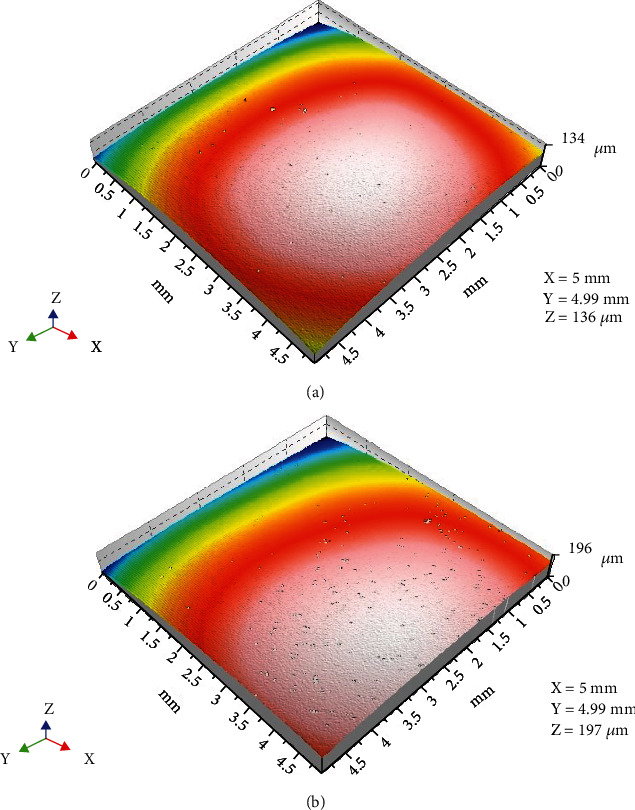
3D surface profile image of Tetric EvoCeram: (a) before chewing simulation and (b) after chewing simulation.

**Figure 5:**
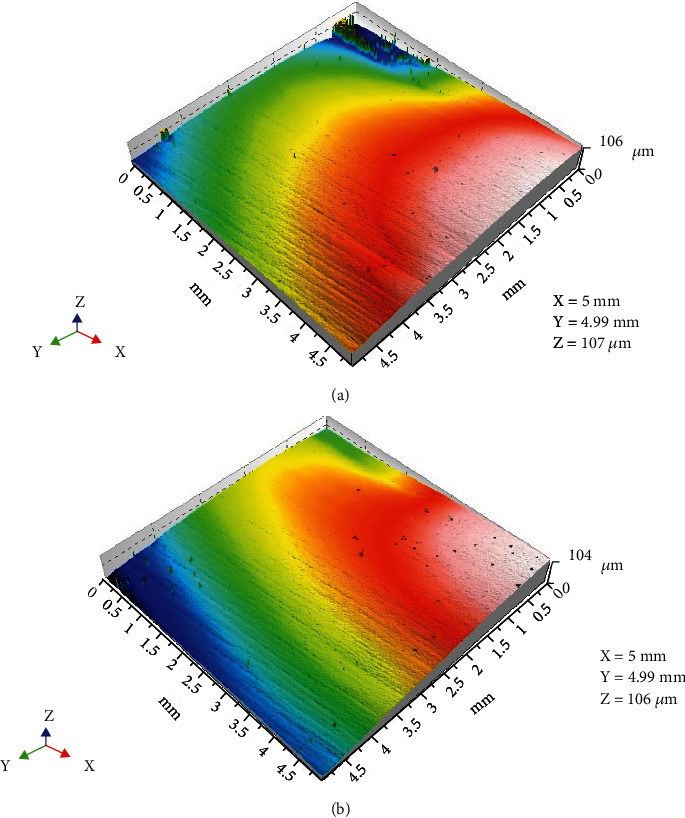
3D surface profile image of Sinfony: (a) before chewing simulation and (b) after chewing simulation.

3D surface profile images for Tetric EvoCeram showed an initial smooth surface while a pitted surface was apparent after chewing simulation (Figure 4). On the other hand, there were no apparent surface differences before and after chewing simulation for Sinfony as shown in 3D surface profile images (Figure 5).

## 4. Discussion

Monolithic zirconia restorations are marketed by manufacturers as antagonist friendly. They have been introduced to overcome the problem of chipping or breaking of veneering ceramic. In the present study, the surface roughness of two resin-composite materials (direct and indirect) was evaluated before and after simulated chewing when opposed by monolithic zirconia. The direct nanohybrid resin-composite material (Tetric EvoCeram) had a smoother surface than the microhybrid indirect material (Sinfony) before and after simulated chewing. Moreover, surface roughness parameters for both materials were generally higher after simulated chewing, and this reflects the occurrence of surface damage which was more apparent for the direct resin-composite (Tetric EvoCeram) compared to the indirect material (Sinfony).

The surface roughness parameters assessed in the present study were *Sa*, *Sq*, *Sv*, and *Sp*. *Sa* is one of the most used parameters to quantify surface texture, but it has drawbacks that may give misleading results as *Sa* represents the magnitude of the surface height, not its spatial distribution. In addition, different surface textures may give similar *Sa* values although they function differently. Assessment of other parameters in addition to *Sa* is therefore necessary to obtain a more precise indication of surface roughness which can be used as a reference or primary measurement.

Based on the results obtained in the current study, the values of the surface roughness parameter (*Sa*) of both materials before and after chewing simulation were clinically acceptable as they were much below the proposed threshold in the literature (200 nm) for plaque retention on the materials' surfaces [29]. The initial assessment of both composite materials after polishing revealed that Sinfony was the material that exhibited significantly higher initial roughness compared to Tetric EvoCeram in all parameters indicating that this material gives higher peaks and deeper valleys, hence increased roughness; accordingly, the first null hypothesis was rejected. This can be explained by the smaller average size of the inorganic fillers incorporated in the matrix of Tetric EvoCeram (<550 nm) compared to that of Sinfony (500-700 nm) resulting in superior polishability of the former material. This finding is in agreement with other studies which finds nanohybrid resin-composite to be smoother than microhybrid resin after different polishing procedures [30]. After chewing simulation, the surface roughness parameters for both materials increased. The difference between surface roughness parameters before and after simulated chewing was significant for the parameters (*Sa*) and (*Sv*) for Tetric EvoCeram, but no significant differences were shown for Sinfony; thus, the second null hypothesis was partially rejected. This is consistent with the findings of a previous study that showed a significant increase in roughness of micro- and nanohybrid direct resin-composites after simulated tooth brushing [30]. The surface roughness parameters were still higher for Sinfony compared to Tetric EvoCeram after chewing simulation (except *Sv*) with a significant difference noted between the two materials in the parameters (*Sp*) and (*Sq*). However, when comparing the extent of change in surface roughness parameters before and after simulated chewing for the two materials, Sinfony exhibited a lower percentage change in all roughness parameters except (*Sq*) indicating a surface more resistant to the effects of chewing compared to Tetric EvoCeram. A higher change in roughness was expected for Sinfony based on its lower filler content (40% by weight) when compared to Tetric EvoCeram (83% by weight) since the resin matrix is usually the component most susceptible to wear. However, in the current study, Tetric EvoCeram showed higher percentage increase in surface roughness. This could be explained by displacement of matrix constituents rather than actual substance loss of Sinfony under chewing simulation. Moreover, the difference in curing technique of the resin matrix can affect the wear behaviour of the resin. As an indirect resin-composite, Sinfony was subjected to two light curing cycles (initial curing and secondary curing) where the secondary curing is carried out in a special device where all surfaces are exposed to curing light for a total time of 15 min. This could have resulted in Sinfony having a resin matrix with greater degree of conversion and subsequently higher mechanical properties and wear resistance compared to the direct resin-composite material Tetric EvoCeram [31].

Although several studies have investigated surface roughness of dental materials, it is difficult to compare the results due to differences in experimental protocols, materials used, and methodology. The use of different antagonists, number of cycles, applied load, thermocycling, and scanning devices may well yield different results. Future research to evaluate the wear volume using three-body wear with anatomical shaped samples and thermocycling with the assessment of changes in the surface of the antagonist can provide better information about the material behaviour to guarantee long-term clinical susses. One of the limitations of the current study is the lack of using opposing human enamel surfaces to act as a blank control group. Also, assessing the effect on surface roughness when resin-composite materials are opposed by human enamel could have been assessed to compare the effect of zirconia and human enamel as antagonists to resin-composite. Although these interactions have been widely assessed and were not among the objectives of the current study, they should be considered when designing future studies.

Based on the results of the current study, both tested resin-composite materials would demonstrate satisfactory surface roughness before and after chewing in terms of the roughness threshold proposed for biofilm accumulation on dental restorations. However, it could be suggested that the use of indirect lab-made resin-composite restorations on teeth opposed by monolithic zirconia restorations would me more favourable over direct resin-composite restorations in terms of surface resistance to damage and change in roughness under the effect of mastication.

## 5. Conclusions

Within the limitations of the current study, the following conclusions could be drawn:

1. The direct nanohybrid resin-composite material (Tetric EvoCeram) had a smoother surface than the microhybrid indirect material (Sinfony) before and after simulated chewing.
2. Surface roughness parameters for both materials were generally higher after simulated chewing; this reflects the occurrence of surface damage which was more apparent for the direct resin-composite (Tetric EvoCeram) compared to the indirect material (Sinfony).

## Figures and Tables

**Table 1:** The composition and manufacturer details of the tested materials.

	Trade name	Type	Composition	Batch number/shade	Manufacturer
Tested resin-composite materials	Tetric EvoCeram	Nanohybrid, direct resin-composite	The matrix is 17–18 wt% dimethacrylate resin. Fillers contain barium glass, ytterbium trifluoride, mixed oxides, and prepolymer (82–83 wt%). The mean filler particle size is <550 nm.	590313/shade A2	Ivoclar Vivadent limited, Enderby, Leicester, UK
	Sinfony	Microhybrid, indirect lab resin-composite	The matrix comprises 48 wt% mixture of aliphatic and cycloaliphatic monomers. Fillers: 40 wt% strontium aluminium borosilicate glass of mean particle diameter 0.5–0.7 *μ*m as macrofiller and 5 wt% pyrogenic silica as microfiller.	049310/shade E2	3M ESPE AG, Seefeld, Germany
The antagonist zirconia ceramic	Lava plus	Second generation high translucency zirconia	3 mol% yttria partially stabilized tetragonal zirconia polycrystal (<15% cubic phase in zirconia, ≤0.5% Al_2_O_3_).	357797/shade A3	3M ESPE, St Paul, MN, USA

**Table 2:** The chewing simulator test parameters.

Parameters	
Weight per specimen	5 kg
Vertical movement	2.5 mm
Horizontal movement	0.7 mm
Vertical speed	60 mm/s
Horizontal speed	40 mm/s
Number of cycles	250,000
Cycle frequency	1.6 Hz
Kinetic energy	2.250 × 10^–6^

**Table 3:** Amplitude parameters used in the study to assess surface roughness.

Parameters	Description
*Sa*	Arithmetic mean deviation within the sample surface area
*Sq*	Root mean square deviation within the sample surface area
*Sp*	Maximum peak height or the maximum height of the profile above the mean line within the assessment surface area
*Sv*	Maximum valley depth or the maximum depth of the valley below the mean line within the assessment area

**Table 4:** The percentage increase of roughness after chewing simulation for both resin-composites (Tetric EvoCeram, Sinfony).

Surface roughness parameters	% Increase of roughness		*p* value
	Tetric EvoCeram	Sinfony	
*Sa*	34.06	11.64	0.140
*Sp*	36.14	6.45	0.205
*Sq*	17.35	21.64	0.840
*Sv*	42.23	-1.90	**0.006**∗

^∗^Significance set at *p* ≤ 0.05.

## Data Availability

The data used to support the findings of this study are included within the article.
